# Comparison of T1/2 Tongue Carcinoma with or without Radial Forearm Flap Reconstruction Regarding Post-Therapeutic Function, Survival, and Gender

**DOI:** 10.3390/cancers15061885

**Published:** 2023-03-21

**Authors:** Katharina El-Shabrawi, Katharina Storck, Jochen Weitz, Klaus-Dietrich Wolff, Andreas Knopf

**Affiliations:** 1 Department of Otorhinolaryngology, Head and Neck Surgery, Faculty of Medicine, Medical Centre, University of Freiburg, 79106 Freiburg, Germany; 2 Department of Otorhinolaryngology, Head and Neck Surgery, Klinikum rechts der Isar, Technical University, 81675 Munich, Germany; 3 Clinic and Policlinic for Oro-Maxillofacial Surgery, Klinikum rechts der Isar, Technical University, 81675 Munich, Germany; 4 Department of Oral and Maxillofacial Surgery, Josefinum, Augsburg and Private Practice Oral and Maxillofacial Surgery im Pferseepark, 86157 Augsburg, Germany

**Keywords:** tongue cancer, primary closure, flap reconstruction, functional outcome, survival outcome, gender distribution

## Abstract

**Simple Summary:**

Surgical therapy for tongue carcinoma is challenging due to the various important functions of the tongue. In order to compensate for loss of tongue tissue and function, flap reconstruction has been firmly established. Interestingly, a large number of early-stage tongue cancer receive flap reconstruction despite minor tissue loss. This study aims to investigate functional and survival differences as well as epidemiologic characteristics in tongue carcinoma patients with or without flap reconstruction. Our retrospective and prospective analyses show no significant survival or functional differences between the groups with or without flap reconstruction. Still, we were able to demonstrate that the possibility of flap reconstruction leads to a more generous tumor resection, less frequent presence of close margin, and subsequently less frequent use of toxic adjuvant therapy regimens. Moreover, for the first time, a significantly higher female ratio could be depicted in the reconstruction group (*p* = 0.02). These findings suggest that, apart from oncologic and functional factors, proportional aspects should be taken into consideration for future decisions on the optimal reconstruction method.

**Abstract:**

Background: Flap reconstruction is commonly used in advanced tongue carcinoma in order to compensate for the loss of tongue tissue and function. Surprisingly, a large number of reconstructed early-stage tongue cancer can be found. Survival or functional benefits in these cases remain unclear. Methods: A retrospective data analysis of 384 surgically treated tongue carcinoma patients was conducted aiming to find epidemiologic and survival differences between patients with (*n* = 158) or without flap reconstruction (*n* = 226). A prospective functional analysis was performed on 55 early-stage tongue cancer patients, 33 without and 22 with radial-forearm flap reconstruction, focusing on post-therapeutic swallowing function as the primary endpoint, speech as the secondary endpoint, xerostomia, quality of life, and mouth opening. Results: Consistent with the current literature, we demonstrated the significantly more frequent use of flap grafts in advanced tongue carcinomas. For the first time, we depicted a higher female ratio in the reconstructed group (*p* = 0.02). There were no significant differences in survival or functional outcomes between the groups. The none-reconstructed group showed more frequent use of adjuvant C/RT despite presenting fewer N+ stages. Conclusions: The higher female ratio in the reconstruction group is plausible due to the anatomically smaller oral cavity and relatively larger carcinoma in women. A higher presence of close margins in the none-reconstruction group may explain the more frequent use of adjuvant C/RT. Since we found no survival or functional differences between the groups, we propose a critical approach toward flap reconstruction in T1/2 tongue carcinoma. At the same time, proportional aspects and adequate resection margins should be taken into account.

## 1. Introduction

Tongue cancer represents one of the most common subtypes of oral cancer, with the majority of it being squamous cell cancer [1]. Annually, there are estimated 354,900 new cases and 177,400 deaths associated with oral cancer worldwide [2]. In addition, an increasing incidence of tongue cancer has been reported over the last few years [3]. The two major risk factors for developing tongue cancer are tobacco and alcohol abuse. Despite its growing significance in the development of oropharyngeal cancer, human papilloma virus (HPV) does not show major relevance in the etiology of tongue cancer [4]. Apart from radiation and chemotherapy, primary surgical resection holds the greatest importance in the therapy of tongue cancer. In a randomized, prospective trial, Iyer et al. showed the significant advantage of surgery compared to primary chemo/radiotherapy (C/RT) regarding survival in oral cancer patients [5]. While surgical treatment remains the first-choice therapy for tongue cancer, a higher focus on its functional outcome must be established. The anatomical reduction in tongue tissue evolves into a functional loss and impairs essential abilities such as speaking, swallowing, and eating, as well as the overall quality of life [6,7]. Furthermore, deteriorated tongue functionality can lead to a decline in survival through complications such as aspiration. For this reason, especially for wide tumor resections in advanced tumor stages, flap reconstruction has been firmly established in order to compensate for the loss of tongue volume and function [6]. However, for early-stage tongue cancer (T1/2), there is an inconsistent opinion on the necessity of flap reconstruction [8,9]. In the literature, there is poor data concerning the functional or survival benefits of performing flap reconstruction after smaller resections of tongue tissue. However, a large number of flap-reconstructed early-stage tongue cancers can be observed. Oncologically, the possibility of flap reconstruction during tumor resection might allow an extension of safety margins and thus better survival outcomes. Consequently, the higher rate of tumor-free margins could result in less use of adjuvant therapy in nodal-negative patients. Considering postoperative functionality, flap reconstruction could improve tongue mobility and thus have a positive effect on speech and swallowing function. These aspects prompted us to investigate not only functional but also survival outcomes after partial glossectomy in patients with or without flap reconstruction. We performed a retrospective analysis on a cohort of 384 surgically treated tongue cancer patients regarding therapeutic modalities and survival as well as epidemiological characteristics. Based on this retrospectively analyzed cohort, we recruited patients with surgically treated T1 and T2 tongue carcinomas for further prospective analyses of their functional outcomes. To receive conclusive results, we defined clear inclusion criteria and recruited only patients in tumor stages T1 and T2 with carcinomas of only the mobile tongue to exclude the possible involvement of the floor of the mouth. To form comparable cohorts, only patients who received reconstruction with or without radial-forearm flaps were included. Postoperative functionality was assessed in swallowing as the primary study endpoint, speech as the secondary endpoint, sicca symptoms, mouth-opening, and overall quality of life. The null hypothesis was that flap reconstruction improves functional outcomes.

## 2. Materials and Methods

### 2.1. Retrospective Analysis

To get a rough breakdown of patients’ epidemiology, therapeutic regimes, and particularly the type of tongue reconstruction, a total of 384 patients with surgically treated tongue carcinoma were analyzed retrospectively. Patients from both the department of otorhinolaryngology and the clinic for oro-maxillofacial surgery were included. Epidemiological data, e.g., age, gender, therapy, reconstruction method, as well as survival data (death/loss to follow-up), were analyzed according to the UICC manual, 7th edition. Patients with a known history of head and neck cancer were excluded from the study. Retrospective data collection and analysis were confirmed by the local ethical committee.

### 2.2. Patient Selection

A homogeneous sub-cohort of T1/2 cancer patients was chosen for further functional assessment. To avoid surgical bias, only patients with T1/2 cancer of the mobile tongue and without the involvement of the floor of the mouth were included. Patients were divided into two groups: patients with and without radial forearm flap reconstruction (RFF).

**Figure 1:**
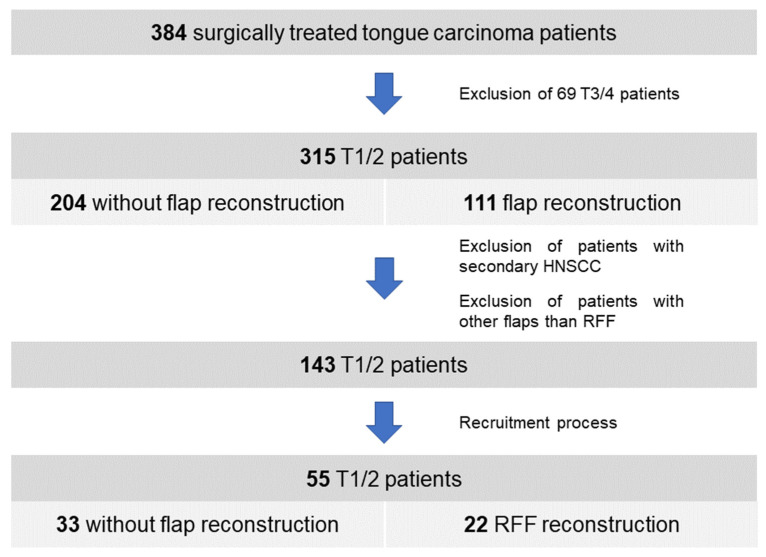
Patient selection.

From the 384 retrospectively identified patients, there were 204 T1/2 patients without flap reconstruction and 111 T1/2 patients who received any flap reconstruction. To receive reliable results regarding swallowing function, we excluded all patients with secondary tumors in the head and neck area. To build comparable groups, we additionally excluded all patients who received other flaps than RFF. Finally, 143 T1/2 patients without flap reconstruction or radial forearm flap reconstruction remained. RFF was selected because it is considered the first choice for small defects of the tongue [10]. A total number of 55 patients who had undergone partial glossectomy agreed to participate in the study, the other 88 patients could not be recruited due to residential distance, missing contact information, personal reasons, their medical condition, or were already deceased (Figure 1). Informed consent was obtained from all 55 patients.

Reconstruction with an RFF was performed on 22 patients. Thirty-three patients underwent surgery with primary closure or healing by secondary intention. The date of surgical intervention and the date of functional analysis were defined and calculated as delta therapy analysis in months. Additionally, clinical parameters on age, sex, height, and weight, TNM-staging (referring to the UICC 7th edition), grading, and treatment modalities, as well as personal risk factors such as nicotine consumption in pack years and alcohol consumption in quantity and quality, were collected. Furthermore, histopathological data on maximum tumor diameter, maximum depth of penetration, minimal tumor-free margin, as well as tumor-free margins at first pass or by follow-up resection were gathered retrospectively. At least two experienced pathologists histologically reviewed all tumor samples.

The local ethical committee approved the study (289/16S).

### 2.3. Swallowing Assessment

To assess the extent of dysphagia, patients completed the 100 mL water swallowing test (WST) [11], which portrays the primary endpoint of the study. The WST previously proved to be a valuable tool to assess post-treatment swallowing performance in head and neck cancer patients [12,13]. In addition, swallowing dysfunction was determined by the M.D. Anderson Dysphagia Inventory (MDADI) [14], which contained 20 dysphagia-related questions and was self-completed by the patients. A score between 0 and 100 could be achieved, with lower scores indicating higher levels of dysphagia. Moreover, a score for changes in eating habits was developed based on the Toxicity Criteria of the Radiation Therapy Oncology Group (RTOG) and the European Organization for Research and Treatment of Cancer (EORTC) [15]. Patients were categorized into five different groups by the examiner according to their dietary changes (Table 1).

### 2.4. Speech Assessment

Speech problems were evaluated using the Speech Handicap Index (SHI) [16], which was self-completed by the patients. The questionnaire consisted of 30 items dealing with daily impairments in social interactions. A score between 0 (no problems) and 120 (high grade of speech problems) could be obtained.

### 2.5. Xerostomia Assessment

Mouth dryness was assessed in patients performing the Saxon Test [17]. To quantify saliva production, patients insalivated a 5 × 5 cm sterile sponge for 2 min. The sponge was weighed before and after salivation. Additionally, all patients completed the Visual Analogue Scale xerostomia questionnaire (VAS) for salivary dysfunction [18], which contained eight items dealing with problems caused by mouth dryness. For each item, a symptom severity scale between 0 and 100 was calculated; higher levels indicating more symptoms. The test included the following questions:

Q1. Rate your difficulty in talking due to dryness

Q2. Rate your difficulty in chewing due to dryness

Q3. Rate your difficulty in swallowing solid food due to dryness

Q4. Rate the frequency of your sleeping problems due to dryness

Q5. Rate your mouth or throat dryness when eating food

Q6. Rate your mouth or throat dryness while not eating

Q7. Rate the frequency of sipping liquids to aid swallowing food

Q8. Rate the frequency of sipping liquids for oral comfort when not eating

### 2.6. Mouth-Opening

Due to surgery and radiotherapy in tongue cancer patients, problems with mouth opening are frequently reported [19]. We assessed mouth opening in all patients as the maximal distance from the upper alveolar ridge to the lower alveolar ridge using a measuring compass. Measurements were taken in millimeters. Furthermore, we determined the Mallampati score in all patients.

### 2.7. Quality of Life Assessment

In order to assess the physical and psychosocial quality of life after cancer surgery, patients completed the Head and Neck module of the EORTC Quality of Life Questionnaire (QLQ-H&N35) [20]. The questionnaire contained 35 items and portrayed frequently reported health and lifestyle changes in patients diagnosed with head and neck cancer face, including the categories of pain, swallowing, teeth problems, problems with opening mouth, mouth dryness, sticky saliva, loss of senses, coughing, speech problems, feeling ill, social eating, social contact, sexuality, use of pain killers, use of oral supplements or a feeding tube, as well as weight loss and weight gain. A symptom score between 0 (= no symptoms) and 100 was calculated for each category.

### 2.8. Statistical Analysis

Statistical analysis of the data obtained was performed with the Statistical Package for the Social Sciences (SPSS Inc., Chicago, IL, USA). Continuous variables were represented by the arithmetic mean and the corresponding standard deviation. Categorical variables were represented by absolute and relative frequencies. Group comparisons between reconstructed and none-reconstructed patients were performed for all retrospectively gathered data as well as for all prospectively assessed functional results, with the WST being the primary study endpoint. Group comparisons of categorical variables were performed using the chi-square test or, for small data sets, the Fisher exact test. For continuous variables, the unpaired Student’s t-test was applied. Survival rates were defined from the date of surgical intervention and were calculated and illustrated using the Kaplan–Meier method. Differences between survival rates were assessed using the log-rank test. A *p*-value of <0.05 was defined as significant in all statistical tests.

## 3. Results

### 3.1. Retrospective Analysis

**Figure 2:**
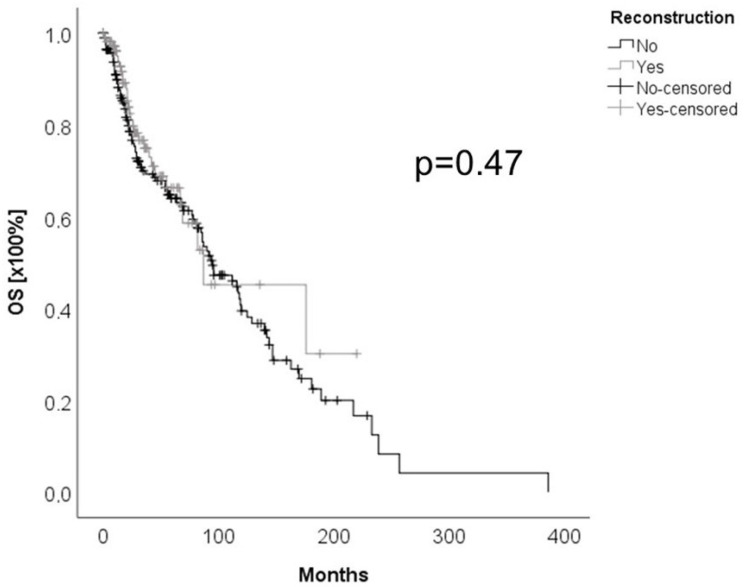
Overall survival (OS) for T1/2 carcinoma comparing primary closure and flap reconstruction.

From a total of 384 surgically treated T1–4 tongue carcinoma patients, 226 patients received primary closure and 158 underwent flap reconstruction. One hundred and four patients received reconstruction using a radial-forearm flap. Regarding T-status, the none-reconstruction and reconstruction groups showed a distribution of 51% vs. 32% of T1, 39% vs. 38% of T2, 6% vs. 22% of T3, and 4% vs. 8% of T4 cases. Both groups were compared, and there were significantly more cases of advanced T-status in the reconstruction group (*p* < 0.0001). Subsequently, we depicted significantly more cases of advanced N-status and positive R-status in the reconstruction group (*p* = 0.03; *p* = 0.02). The TNM status is summarized in Table 2. Adjuvant C/RT was significantly more often performed in the reconstruction group (*p* < 0.0001). Interestingly, we found a significantly higher female ratio (26% vs. 38%) in the reconstruction group (*p* = 0.02). Survival analysis was performed on T1 and T2 cases. Analyses showed a mean overall survival (OS) of 116 months in the none-reconstruction group and 118 months in the reconstruction group. There was no significant advantage regarding OS from performing flap reconstruction (*p* = 0.47, Figure 2).

To identify whether the significantly higher female ratio in the reconstruction group was due to an also advanced T-status in women, we analyzed the distribution of T status among men and women in the reconstructed patients. Analyses showed a significantly higher proportion of T3–4 status in men (34% vs. 21%; *p* = 0.005) among all reconstructed patients (Table 3).

In order to specify the results for early tumor stages, we performed an additional data analysis for T1 and T2 stages only. Here, there was no statistically significant difference for N, M, and R stages between the reconstructed and non-reconstructed tongue carcinomas. Regarding therapy regimens, adjuvant therapy was used more frequently in the reconstruction group, which can be attributed to the correspondingly higher presence of N+ in this group (Table 4).

### 3.2. Epidemiology of the Prospective Functionally Analyzed Cohort

To form comparable groups for the assessment of functional outcomes in early-stage tongue cancer, we excluded all patients who received different flaps than the RFF. Finally, a total of 55 patients diagnosed with tongue cancer in tumor stages T1 and T2 participated in this study, comprising 33 patients who underwent partial glossectomy without reconstruction and 22 patients who underwent partial glossectomy with RFF reconstruction. At this point, it should be taken into account that biases with regard to patient selection are possible due to the small number of cases. Patients’ treatment decision was made after the tumor board consensus and recommendation of head and neck surgeons. The mean duration between surgery and functional analysis was 78 months and 66 months, respectively, without statistically significant differences between the groups (*p* = 0.49, Table 5). The mean age of patients at initial diagnosis was 49 years and 51 years. A higher female ratio (15% vs. 36%) was found in the RFF-reconstruction group, although it could not be regarded as significant (*p* = 0.09). The height-to-weight ratio did not differ significantly between the groups (*p* = 0.06). Only one patient in the RFF-reconstruction group required a gastral tube; none required a permanent tracheostomy.

A distribution of T1 (67–68%) and T2 (32–33%) was evenly reported in both groups, as well as a majority of N0-status (82% and 77%). When compared to the RFF-reconstruction group, the none-reconstruction group showed a significantly advanced grading, with 89% of patients being diagnosed with G2 or G3 (*p* = 0.016). In both groups, the maximum tumor diameter ranged from 16 to 17 mm and the maximum depth of penetration from 8 to 9 mm. The minimally achieved tumor-free margin amounted to 4 mm in both groups. R0 resection on the main sample could be achieved in 67% of cases in the none-reconstruction group and in 77% of cases in the RFF-reconstruction group (*p* = 0.45). Other patients underwent R0 resection during the same surgery by follow-up resection. In this study, there was only one patient in the RFF-reconstruction group who was diagnosed with R1 resection in the final histology. The patient refused further surgical procedures and underwent adjuvant treatment.

All patients received a neck dissection. In both groups, the majority of patients did not undergo an additional adjuvant therapy, referring to nodal negativity and R0 resection. Additional radiotherapy was performed on 33% and 14%, and additional chemo-radiotherapy was performed on 6% and 14%, respectively (*p* = 0.81). Subsequently, seven patients without RFF underwent adjuvant treatment escalation due to close margins, while only one patient in the RFF group underwent adjuvant treatment escalation referring to R1 status. However, this tendency did not achieve statistical significance (*p* = 0.09).

There were slightly fewer patients with prior or active use of nicotine in the none-reconstruction group, comprising 55% and 46% of none-smokers (Table 6). The average smoking time ranged from 17py in the none-reconstruction group to 15py in the RFF group. A non-significant higher consumption of alcohol could be depicted in the none-reconstruction group, showing an average daily alcohol consumption of 615 mL or 397 mL respectively (*p* = 0.46). Drinking habits, considering active, prior, or none alcohol consumption, did not differ significantly between the groups. In both groups, the patients’ majority continued alcohol consumption after surgery (42% and 73%). In both groups, the most common liquid consumed was beer.

### 3.3. Swallowing

**Figure 3:**
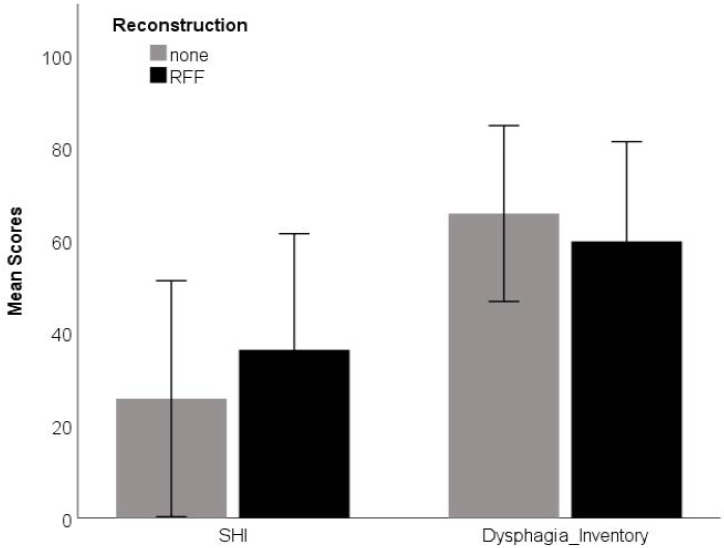
Results of the Speech Handicap Index (SHI) and the MD Anderson Dysphagia Inventory (MDADI) compared. A low symptom score in the SHI represents fewer problems with speech. A low score in the MDADI indicates greater difficulties in swallowing function.

The primary endpoint of this study was swallowing function determined through the 100 mL water swallowing test (WST). The mean amount of water swallowed per second in patients with primary closure was 17.89 mL/s. Patients receiving an RFF reconstruction swallowed 15.96 mL/s. In this objective assessment of patients’ swallowing function, there were no statistically significant differences between both groups (*p* = 0.39, Table 7). Additionally, no significant differences in nasal reflux (18% vs. 5%; *p* = 0.1) or in the RTOG dysphagia score (0.44 vs. 0.86; *p* = 0.09) were found. As a subjective assessment of patients swallowing function, the MD Anderson dysphagia inventory showed a remaining swallowing function range of 60–65/100 in both groups (Figure 3). No significant differences between the groups were reported in this test (*p* = 0.28).

### 3.4. Speech

The Speech Handicap Index represented the secondary endpoint of this study and depicted patients’ subjective speech issues. The mean total SHI score ranged from 25 points in the none-reconstruction group to 37 points in the RFF-reconstruction group, showing fewer speaking problems in the none-reconstruction group (Figure 3). There were no significant differences between the groups (*p* = 0.14).

### 3.5. Xerostomia

**Figure 4:**
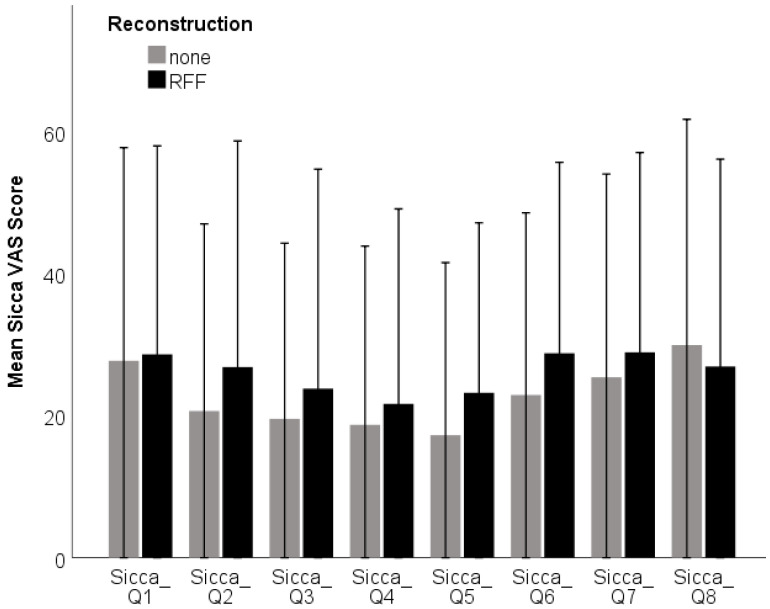
Results of the Sicca VAS score.

Saliva production in the Saxon test ranged from 2.07 g/2 min in the none-reconstruction group to 1.87 g/2 min in the RFF-reconstruction group. The Sicca VAS score revealed symptom scores from 18% (Q5) to 30% (Q8) in the none-reconstruction group and 22% (Q4) to 29% (Q1) in the RFF-reconstruction group (Figure 4). In both the objective and subjective tests, no significant differences were found between the groups (*p* = 0.53; *p* = 0.71).

### 3.6. Mouth-Opening

The maxilla-mandible (gingiva-to-gingiva) distance ranged from 60.34 mm in the none-reconstruction group to 61.14 mm in the RFF-reconstruction group. The none-reconstruction group showed a Mallampati score of 2.53, and the RFF-reconstruction group had a score of 2.36, respectively. In both tests, there were no statistically significant differences between the groups (*p* = 0.81; *p* = 0.62).

### 3.7. Quality of Life

**Figure 5:**
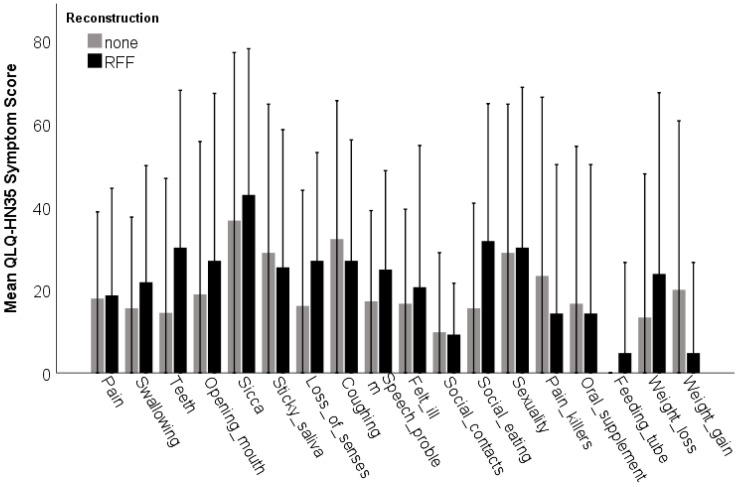
Results of the EORTC QLQ-HN35 for primary closure and RFF reconstruction compared.

The most severe symptom reported in the QLQ-HN35 was mouth dryness in both groups, followed by coughing in the none-reconstruction group and social eating in the RFF-reconstruction group. On any of the symptom scales, there were no statistically significant differences found between the groups. Figure 5 illustrates all 18 symptom scales for both groups.

## 4. Discussion

Flap reconstruction is firmly established in the advanced stages of tongue carcinomas, aiming to restore volume and preserve tongue mobility [6,21]. Our retrospective analyses of the large cohort of 384 tongue cancer patients showed significantly more cases of advanced T- and N-status as well as positive R-status in the reconstruction group when compared to the none-reconstruction group. In addition, escalation of therapy via adjuvant C/RT was depicted more often in the reconstruction group, which is coherent with advanced T-, N-, and positive R-status. These findings are consistent with the current literature [22,23,24]. Interestingly, our retrospective analyses also depicted a large amount of reconstructed early-stage tongue cancer. In fact, we were able to denote that the majority of reconstructed patients were diagnosed with either T1 (32%) or T2 (38%). This observation suggests either a functional or a survival benefit from performing flap reconstruction and prompted us to further investigate these parameters.

From a surgical point of view, survival can be improved by extending the distance between the tumor and the resection margin. Several studies have shown that the margin size and achieving R0 on the main sample correlate with significant improvements in recurrence-free intervals (RFI) and thus OS [25,26]. Consequently, the possibility of flap reconstruction might surgically lead to a more generous tumor resection and improve the chances of R0 on the main sample. Through this consideration, reconstruction could contribute to a survival benefit. Analyses of the histopathological data of our functionally examined patients did not show a statistically significant difference concerning R0 on the main sample between the groups. This indicates that sufficient resection can be achieved even without flap reconstruction. These results were confirmed by the analysis of overall survival in the large retrospective group. There were no statistically significant differences regarding OS in the T1 and T2 stages between the none-reconstruction and reconstruction groups (116 vs. 118 months, *p* = 0.47). In contrast, we were able to depict the more frequent use of adjuvant C/RT in the none-reconstructed group when compared to the RFF group (39% vs. 28%), despite the predominant N0 status in both groups (82% and 77%). Indications for adjuvant radiotherapy in T1/2 oral cancer include positive resection margins (R1) or the presence of lymph node metastases (N+) [27]. Lymph node metastases with extracapsular extension (ECE) are an indication of adjuvant chemo-radiotherapy [28]. In the case of close margins, adjuvant radiotherapy is also frequently applied [27]. Since the none-reconstructed group showed fewer R0 resections on the main sample, there are higher chances of close margins in these cases. The higher presence of close margins consequently led to the more frequent use of adjuvant C/RT escalation in the none-reconstruction group, comprising 21% and 5%, respectively. In turn, these results suggest that, due to the possibility of flap reconstruction, a more generous tumor resection was possible in the RFF group, with lower chances of close margins and therefore a less frequent need for adjuvant C/RT and its respective concomitant toxicities. However, this tendency failed to achieve statistical significance in our study (*p* = 0.09). Lu et al. compared margin size and recurrence rate in a large cohort of 347 early-stage tongue carcinoma patients and found that the utilization of flap reconstruction achieved a significantly larger pathologic free margin and had significantly lower recurrence rates [29].

The surgical objective after partial tongue resection should not only include the best oncological outcome but also consider functional aspects. Essential functions such as airway protection, swallowing, and speaking should be safely practicable and maintain an optimal quality of life for the patient. Regarding functionality in advanced tumor stages, Canis et al. compared microvascular flap reconstruction and primary closure in T3 tongue carcinomas and showed significant functional impairments in patients who did not receive flap reconstruction [30]. Our study assessed functionality in T1 and T2 tongue carcinomas in both subjective and objective test batteries. Our results show that there is no significant functional advantage in any field examined. Regarding swallowing, the none-reconstruction group even showed a slightly better mean of water-drinking time, a lower symptom score in the QLQ-HN35, a lower RTOG dysphagia score, and better results in the MD Anderson dysphagia inventory, despite not being statistically significant. Only the presence of nasal reflux was observed to be less frequent in the RFF reconstruction group. A similar trend could be observed in the subjective speech questionnaires: both the QLQ-HN35 symptom score for speech problems and the SHI portrayed slightly more problems with speech in the RFF-reconstruction group, although this could not be regarded as statistically significant. These findings demonstrate that the flap not only serves to provide bulk and improve mobility but can also interfere with processes such as articulation or swallowing and necessitates training. Similar results were obtained in a study by Ji et al. showing significantly worse functional outcomes in articulation, tongue mobility, and speech intelligibility in flap-reconstructed tongue carcinoma patients [6]. A study by Kaur et al. placed the main emphasis on subjective patient satisfaction and found that higher levels were achieved for the primary closure of smaller defects and for flap reconstruction of larger tongue defects [31]. Interestingly, the most important factor regarding the QLQ-HN35 with the highest symptom scores for both groups was mouth dryness, which was also demonstrated to be relevant in the Sicca VAS score. This subjectively present mouth dryness was objectively confirmed in the Saxon test, demonstrating a mean saliva production of <2.75 g/2 min in both groups, which was defined as pathological [17]. A major factor that can cause dry mouth is the use of adjuvant C/RT.

The time range between the end of therapy and functional assessment was 78 months and 66 months, respectively; therefore, this study mainly depicts long-term functional outcomes. In this period of time, other factors might have impacted speech and swallowing function, which must be considered when applying the results for clinical purposes. Still, a comparison considering long-term functional outcomes was possible since the period between therapy and functional assessment did not differ significantly between the groups (*p* = 0.49).

When analyzing our retrospective cohort, we saw a statistically significant higher distribution of women receiving flap reconstruction (*p* = 0.02). To identify whether this was due to an also advanced T-status in women, we analyzed the T-status distribution among reconstructed male and female patients. Surprisingly, analyses showed significantly smaller T status in reconstructed women when compared to men (*p* = 0.005). In conclusion, this shows that women received flap reconstruction despite having a smaller T-status. Anatomically, women tend to have smaller oral cavities and less tongue tissue, which indicates that T1 or T2 tongue carcinomas in women are proportionally larger than in men. Therefore, the more frequent usage of flap reconstruction is conclusive. Generally, these findings suggest that not only the tumor size should be taken into consideration but also its proportion to the remaining tongue tissue and protection of the mandible. A uniform classification system for tongue reconstruction does not yet exist. However, possible solutions have been pointed out in various studies so far. Mannelli et al. proposed a strategic approach for surgery of different types of tongue defects where a specific reconstruction algorithm is available for each type of defined tongue defect [23]. Ansarin et al. proposed a similar classification system that is based on the anatomical and functional components of the tongue and the spread routes of tongue cancer [32]. A proportional concept with consideration of the actual size of the tongue and oral cavity is missing in both studies. Given our results, the distance between the tumor and the tongue midline, for instance, could serve as an indicator of the relative volume loss and consequently be used as a criterion for flap reconstruction. Our research revealed no other previous study describing differences in tongue reconstruction in women.

To further determine whether N or R status exerted an influence on the decision to perform flap reconstruction, we specifically analyzed their distribution in early tumor stages of tongue carcinoma in the retrospective cohort. Again, there was no significant difference between the groups. This underlines the fact that the decision to perform flap reconstruction depends individually on the local expertise of the surgeon.

Apart from fitting the proportionally perfect flap, performing microvascular flap reconstruction also involves the management and training of non-innervated tissue, which is crucial for abilities such as speaking and eating. Furthermore, RFF reconstruction involves the loss of the radial artery at the donor site and possible complications such as necrosis of the flap due to vascular anastomosis insufficiency [33,34]. A major advantage when performing primary closure of the tongue is the shorter operative time and thus lower risk of delirium, more likely avoidance of tracheostomy, and no or short intensive care duration.

To our knowledge, this is the first study analyzing objective and subjective parameters in surgically treated early-stage tongue cancer, specifically comparing none-reconstruction to RFF-reconstruction, which is one of the strengths of this study. TNM stages, age, and gender were also evenly distributed between the functionally studied groups, which supports the results from the group comparison. At the same time, the small case number of the functionally examined cohort must be taken into account, as must the possible bias in patient selection. Moreover, the long time range between the end of therapy and functional assessment must be considered when applying the results for clinical purposes, as other factors might have impacted speech and swallowing functions during this period. In contrast, the retrospective analysis of 384 surgically treated tongue carcinoma patients provides a broad overview regarding epidemiological differences and treatment regimens. By including patients from the ENT and maxillofacial surgery departments, we were able to form a very heterogeneous collective, which can be well applied to actual clinical practice. In addition to overall survival, it would have been interesting to measure disease-specific survival as well. This could not be conducted because it was not evident from our existing data if death was caused by a tumor diagnosis. In summary, our findings indicate that a more restrained approach to the usage of flap reconstruction in smaller carcinomas of the tongue is favorable and that the loss of tongue tissue proportionally to the remaining tongue volume should be taken into consideration for an optimal functional outcome. For surgical therapy of T3/4 tongue carcinoma, flap reconstruction is undoubtedly recommended.

## 5. Conclusions

Our study demonstrated that there are no statistically significant differences regarding functional and survival outcomes between flap reconstruction and none-reconstruction in early-stage tongue carcinomas. This suggests that the implications of reconstructing T1 and T2 tongue carcinomas should be deeply evaluated beforehand, taking possible complications and necessary training into consideration. At the same time, we showed that the possibility of flap reconstruction leads to a more generous surgical resection, less frequent presence of close margin, and subsequently less frequent use of toxic adjuvant therapy regimens. Furthermore, we demonstrated for the first time that women were significantly more likely to be reconstructed by flap surgery, even when presenting a smaller T-status. This indicates that reconstruction cannot be determined by the tumor size alone but requires a proportional approach based on existing anatomical circumstances. Future research is needed to identify and develop clear guidelines for the usage of flap reconstruction in early-stage carcinomas of the tongue.

## Figures and Tables

**Table 1:** Dietary changes.

Scheme	Dietary Changes
0	No changes
1	Mild dysphagia with slight changes of eating habits (mild diet), slight difficulties in swallowing solid food.
2	Moderate dysphagia with necessary changes of eating habits to pureed or liquid food, solid food can’t be swallowed.
3	Severe dysphagia with changes of eating habits to only liquid food.
4	Complete obstruction, nutrition requires N-G feeding tube, i.v. fluids or hyperalimentation

**Table 2:** Retrospective data.

	No Reconstruction	Reconstruction	*p*-Value
**N**	226	158	
**Gender, *n*** (**%**)			0.02
Male: Female	167 (74)/59 (26)	98 (62)/60 (38)	
**Age at initial diagnosis** (**years**)			0.99
Mean ± SD (median)	57 ± 14 (56)	57 ± 13 (59)	
**Grading, *n*** (**%**)			0.82
G0	1 (0.4)	0	
G1	34 (15)	19 (12)	
G2	143 (63)	113 (72)	
G3	47 (21)	26 (17)	
G4	1 (0.4)	0	
**T status, *n*** (**%**)			<0.0001
T1	116 (51)	51 (32)	
T2	88 (39)	60 (38)	
T3	14 (6)	34 (22)	
T4	8 (4)	13 (8)	
**N status, *n*** (**%**)			0.03
N0	149 (66)	89 (56)	
N1	37 (16)	23 (15)	
N2a	5 (2)	3 (2)	
N2b	7 (3)	19 (12)	
N2c	12 (5)	10 (6)	
N3	16 (7)	14 (9)	
**M status, *n*** (**%**)			0.32
M0	226 (100)	157 (99)	
M1	0	1 (0.6)	
**R status, *n*** (**%**)			0.02
R0	219 (97)	146 (92)	
R1	7 (3)	6 (4)	
R2	0	1 (0.6)	
Rx	0	5 (3)	
**Therapy**			<0.0001
Surgery	152 (67)	78 (49)	
Surgery + aRT	59 (26)	52 (33)	
Surgery + aCRT	15 (7)	28 (18)	
**Reconstruction**			
RFF	-	104 (66)	
ALT	-	18 (11)	
Perforator	-	8 (5)	
Other	-	24 (15)	
Peroneus	-	4 (3)	

**Table 3:** T status distribution among men and women in reconstructed patients (*n* = 158).

	Male	Female	*p*-Value
**N**	98	60	
**T status, *n*** (**%**)			0.005
T1	25 (26)	26 (43)	
T2	39 (40)	21 (35)	
T3	23 (23)	11 (18)	
T4	11 (11)	2 (3)	

**Table 4:** Calculation of N, M, R stages, therapy, and reconstruction method for T1/2 cases only.

	No Reconstruction	Reconstruction	*p*-Value
**N**	204	111	
**N status, *n*** (**%**)			0.61
N0	143 (70)	74 (67)	
N1	30 (15)	16 (14)	
N2a	4 (2)	-	
N2b	7 (3)	12 (11)	
N2c	6 (3)	4 (4)	
N3	14 (7)	5 (5)	
**M status, *n*** (**%**)			0.32
M0	204 (100)	110 (99)	
M1	-	1 (1)	
**R status, *n*** (**%**)			0.28
R0	197 (97)	106 (96)	
R1	7 (3)	3 (3)	
Rx	-	2 (2)	
**Therapy**			0.07
Surgery	144 (71)	68 (61)	
Surgery + aRT	48 (24)	31 (28)	
Surgery + aCRT	12 (6)	12 (11)	
**Reconstruction**			
RFF	-	66 (60)	
ALT	-	12 (11)	
Perforator	-	8 (7)	
Other	-	21 (19)	
Peroneus	-	4 (4)	

**Table 5:** Epidemiologic data of the functionally analyzed cohort.

	No Reconstruction	RFF Reconstruction	*p*-Value
**N**	33	22	
**Delta therapy analysis** (**months**)			0.49
Mean ± SD (median)	78 ± 66 (52)	66 ± 50 (43)	
**Age at initial diagnosis** (**years**)			0.66
Mean ± SD (median)	49 ± 13 (49)	51 ± 17(50)	
**Gender, *n*** (**%**)			0.09
Male: Female	28 (85)/5 (15)	14 (64)/8 (36)	
**T status, *n*** (**%**)			0.91
T1	22 (67)	15 (68)	
T2	11 (33)	7 (32)	
**Maximum tumor diameter** (**mm**)			0.69
Mean ± SD (median)	16 ± 8 (18)	17 ± 7 (15)	
**Maximum depth of penetration** (**mm**)			0.77
Mean ± SD (median)	8 ± 4 (7)	9 ± 7 (6)	
**N status, *n*** (**%**)			0.64
N0	27 (82)	17 (77)	
N1	3 (9)	3 (14)	
N2a	1 (3)	0	
N2b	1 (3)	0	
N2c	0	1 (5)	
N3	1 (3)	1 (5)	
**M status, *n*** (**%**)			
M0	33 (100)	22 (100)	
M1	0	0	
**Grading, *n*** (**%**)			0.016
G1	4 (12)	5 (23)	
G2	20 (61)	17 (77)	
G3	9 (27)	0	
**R status, *n*** (**%**)			0.33
R0	33 (100)	21 (96)	
R1	0	1 (5)	
**R0 on the main sample**			0.45
No	11 (33)	5 (23)	
Yes	22 (67)	17 (77)	
**Minimal achieved tumor-free margin** (**mm**)			0.89
Mean ± SD (median)	4 ± 3 (4)	4 ± 2 (5)	
**Neck dissection**			0.09
Ipsi-lateral	23 (70)	10 (46)	
Bi-lateral	10 (30)	12 (55)	
**Adjuvant therapy, *n*** (**%**)			0.81
None	20 (61)	16 (73)	
aRT	11 (33)	3 (14)	
aCRT	2 (6)	3 (14)	
**Adjuvant therapy escalation, *n*** (**%**)			0.09
No	26 (79)	21 (95)	
Yes	7 (21)	1 (5)	

**Table 6:** Noxae.

	No Reconstruction	RFF Reconstruction	*p*-Value
**Nicotine abuse** (**py**)	17	15	0.81
None, *n* (**%**)	17 (52)	10 (46)	0.9
Prior, *n* (**%**)	11 (33)	9 (41)	
Active, *n* (**%**)	5 (15)	3 (14)	
**Alcohol consumption** (**mL/d**)	615	397	0.46
None, *n* (**%**)	9 (27)	5 (23)	0.18
Prior, *n* (**%**)	10 (30)	1 (5)	
Active, *n* (**%**)	14 (42)	16 (73)	
**Liquids**			0.54
None, *n* (**%**)	8 (24)	5 (23)	
Beer, *n* (**%**)	14 (42)	12 (55)	
Wine, *n* (**%**)	9 (27)	4 (18)	
Spirits, *n* (**%**)	2 (6)	1 (5)	

**Table 7:** Functional analyses.

	No Reconstruction	RFF Reconstruction	*p*-Value
**Ratio height to weight**	2.30	2.58	0.06
**Gastral tube, *n*** (**%**)	0	1 (5)	0.33
**Tracheostomy, *n*** (**%**)	0	0	
**Water drinking time** (**mL/s**)			0.39
Mean ± SD (median)	17.89 ± 8.46 (18.13)	15.96 ± 7.39 (15.24)	
**Nasal reflux, *n*** (**%**)	6 (18)	1 (5)	0.10
**RTOG dysphagia score**	0.44	0.86	0.09
**Saxon test** (**g/2 min**)	2.07	1.87	0.53
**Mallampati**	2.53	2.36	0.62
**Maxilla-mandible distance** (**mm**)	60.34	61.14	0.81

## Data Availability

The data can be shared up on request.
